# Kynurenines in the Pathogenesis of Multiple Sclerosis: Therapeutic Perspectives

**DOI:** 10.3390/cells9061564

**Published:** 2020-06-26

**Authors:** Tamás Biernacki, Dániel Sandi, Krisztina Bencsik, László Vécsei

**Affiliations:** 1 Department of Neurology, Faculty of General Medicine, Albert Szent-Györgyi Clinical Centre, University of Szeged, H-6725 Szeged, Hungary; biernacki.tamas@med.u-szeged.hu (T.B.); sandi.daniel@med.u-szeged.hu (D.S.); krisztina.bencsik@invitel.hu (K.B.); 2 MTA—SZTE Neuroscience Research Group, H-6725 Szeged, Hungary; 3 Interdisciplinary Excellence Center, University of Szeged, H-6720 Szeged, Hungary

**Keywords:** kynurenine pathway, kynurenic acid, oxidative stress, quinolinic acid, N-acetylserotonin, IDO, NAD^+^, multiple sclerosis, laquinimod

## Abstract

Over the past years, an increasing amount of evidence has emerged in support of the kynurenine pathway’s (KP) pivotal role in the pathogenesis of several neurodegenerative, psychiatric, vascular and autoimmune diseases. Different neuroactive metabolites of the KP are known to exert opposite effects on neurons, some being neuroprotective (e.g., picolinic acid, kynurenic acid, and the cofactor nicotinamide adenine dinucleotide), while others are toxic to neurons (e.g., 3-hydroxykynurenine, quinolinic acid). Not only the alterations in the levels of the metabolites but also disturbances in their ratio (quinolinic acid/kynurenic acid) have been reported in several diseases. In addition to the metabolites, the enzymes participating in the KP have been unearthed to be involved in modulation of the immune system, the energetic upkeep of neurons and have been shown to influence redox processes and inflammatory cascades, revealing a sophisticated, intertwined system. This review considers various methods through which enzymes and metabolites of the kynurenine pathway influence the immune system, the roles they play in the pathogenesis of neuroinflammatory diseases based on current evidence with a focus on their involvement in multiple sclerosis, as well as therapeutic approaches.

## 1. Introduction

Even though kynurenic acid was discovered roughly 170 years ago, it was not until the 1970s and 1980s that the kynurenine pathway (KP) sparked substantial interest among neuroscientists. This was due to the discovery that the two major products of the pathway, kynurenic acid (KYNA) and quinolinic acid (QUIN), possess significant, yet opposing effects on various neuronal cells and physiological processes [1]. The KP was found to be responsible for the overwhelming majority (>90%) of peripheric tryptophan (TRP) metabolism [2]. In the early days of kynurenine research, the belief held for a long time that the main purpose of the KP is solely the production of nicotinamide adenine dinucleotide (NAD^+^), a coenzyme already known to be a pivotal molecule in a vast amount of vital biochemical processes including, but not limited to being a key component in several redox reactions and being vital to mitochondrial function [3]. In the past decades, however, significant attention has been directed to the enzymes and metabolites of the KP, after the discovery that an alteration can be found not only in the metabolite levels but in the activity of the enzymes producing them as well in numerous disorders. They have been implicated to play a role in neurodegenerative, psychiatric and developmental diseases, infections, tumors, autism, vascular diseases, allergies, transplant rejections, cancer immunity, immune privilege disorders and also in various autoimmune and neuroinflammatory conditions [4,5,6,7,8,9,10]. Kynurenic acid was the first member of the “kynurenine family” derived from the essential amino acid TRP. KYNA has been intensively studied in the past decades, has turned out to be a potent antagonist of excitatory ionotropic glutamate receptor on both the N-methyl-D-aspartate (NMDA) and glycineB site. On the other hand, the other main derivate of the pathway, quinolinic acid, is a selective agonist of the aforementioned receptor. The activation of the NMDA receptors (NMDAR) results in a cationic influx (Na^+^, Ca^2+^, K^+^) to the cell; the subsequent increase in intracellular Ca^2+^ level activates several downstream signaling pathways and secondary messenger molecules, which ultimately lead to various synaptic alterations. The superfluous activation of the NMDARs, however, causes an excessive inflow of Ca^2+^ ions, eliciting neurotoxicity and cellular damage, which can even induce neuronal cell death. Neuronal damage due to the excitotoxicity caused by excessive NMDAR activation has been linked to a number of neurodegenerative disorders including Huntington’s, Alzheimer’s disease, amyotrophic lateral sclerosis (ALS), and multiple sclerosis (MS) [11]. Additional to the confirmed excitotoxicity mediated by the overactivation of the NMDARs, an increased level of QUIN was reported in the pathogenesis of these diseases as well [4,5,6,7,8,11]. In addition to finding an elevation in QUIN levels, a decreased amount of KYNA was observed in some of these diseases. This raised the question, that not simply the increasement of QUIN is essential to the pathogenesis, but a more complex dysregulation is underlying, causing a shift of the KYNA to QUIN ratio. Nowadays, through the advancement of genetic and molecular diagnostics, we gain an increasing amount of insight into the pathomechanism of the diseases burdening humanity; the KP seems to be a key player in many of them. In this review, following a concise introduction about the KP and its two most well-defined neuroactive metabolites, we aim to bring together recent evidence of their diverse effect on immunoregulatory mechanisms and their involvement in MS with a focus on a potential future therapeutic approach.

## 2. The Production and Metabolism of Kynurenines

Tryptophan is not only one of the most scarcely found amino acid in mammalian organisms (comprising roughly only 1–1.5% of the total protein amino acid content), but is also an essential amino acid for humans and the precursor amine for the synthesis of essential proteins, nicotinic acid, NAD^+^, the neurotransmitter serotonin, N-acetylserotonin (NAS), and melatonin [12]. The metabolism of TRP can occur through two major pathways: the methoxyndole pathway (also known as the serotonin pathway, which accounts for ~5% of the metabolism) and the KP. The KP represents the primary route of metabolism for both the periphery and the central nervous system (CNS), accounting for approximately 95% of the TRP metabolism [13]. The metabolism of TRP is conducted by a chain of enzymes mostly found in glial cells after TRP enters the CNS and passes the blood-brain barrier (BBB) via the non-specific and competitive L-type amino acid transporter [14]. Roughly 10% of the total TRP circulating in the blood is bound to albumin; the rest of the unbound TRP can be transported across the BBB by the aforementioned transporter [3]. After entering the CNS, TRP can enter either the kynurenine pathway or the methoxyndole pathway. 

### 2.1. Kynurenine Pathway

**Figure 1:**
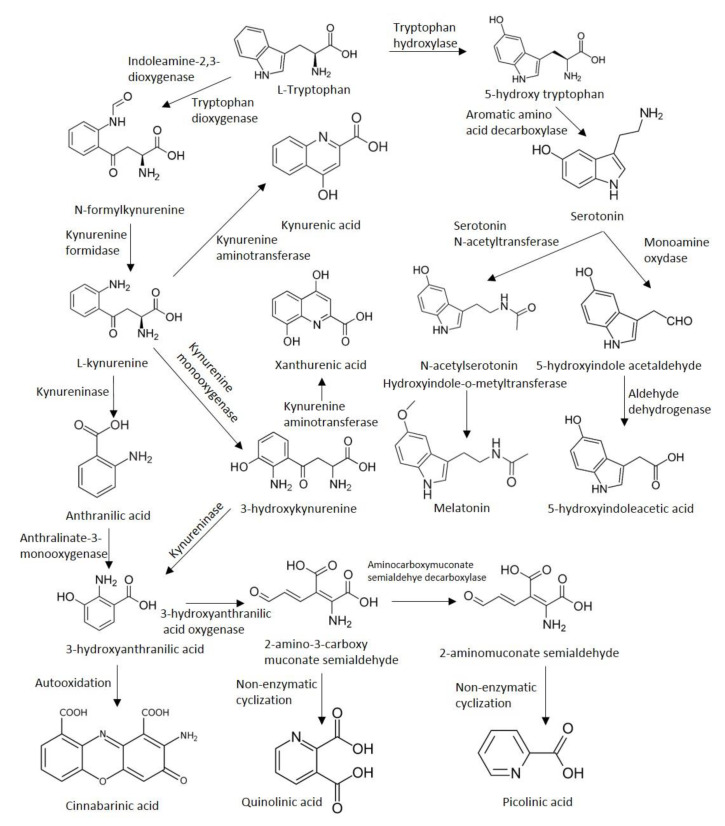
The kynurenine pathway of tryptophan metabolism.

All of the intermediary metabolites of the KP are called kynurenines. The first and rate-limiting step of the KP is the conversion of TRP into N-formyl-l-kynurenine by two enzymes. The first enzyme, indoleamine 2,3-dioxygenase (IDO-1) is most prominently expressed and has the highest activity in dendritic cells [15,16]. It has also been shown to be overexpressed in certain neoplastic tissues [17,18,19] and underexpressed/defective in cases of autoimmune and neuroinflammatory diseases [20,21,22,23,24]. The isoenzyme IDO-2 is mainly expressed in the liver. The second enzyme responsible for the conversion of TRP, tryptophan 2,3-dioxygenase (TDO) is also abundantly expressed in the liver, is responsible for the regulation of the systemic levels of TRP [25]. TDO is also expressed, in lower levels of the CNS: in neurons, endothelial cells, and astrocytes, and similarly to IDO-1, it has been recently found in tumor cells [6,26]. IDO-1 can be potently induced by IFN-γ, TGF-β, Toll-like receptor ligands, and polyamines [27,28,29]. L-kynurenine is formed afterward from N-formyl-l-kynurenine by a formidase. The activity of the two rate-limiting enzymes under physiological circumstances are normally quite low in the CNS [30]. Approximately 60% of metabolism along the KP in the brain is mediated by the uptake of the main metabolite, kynurenine from the blood by glial cells through the neutral amino acid carrier [31,32,33]. The pivotal product, l-kynurenine takes a central position in the pathway, as it can diverge into three very distinct directions from this molecule. L-kynurenine can be further processed by the enzymes kynureninase, kynurenine aminotransferases (KAT; thus far, four isoenzymes have been identified, of which KAT1 and -2 play a capital role in humans [34]), and kynure-nine 3-monooxygenase (KMO). The activity of these enzymes results in the formation of 3-hydroxy-l-kynurenine, KYNA, and anthranilic acid (ANA), respectively. The next molecule, 3-hydroxyanthranilic acid can be either formed from ANA by nonspecific hydroxylases or from 3-hydroxy-l-kynurenine by the action of the kynureninase enzyme. 3-hydroxy-l-kynurenine can be further converted into xanthurenic acid by the KAT enzymes. 3-hydroxyanthranilic acid is further metabolized by 3-hydroxyanthranilate oxidase (3-HAO) into 2-amino-3-carboxymuconate-semialdehyde, an unstable compound. It can be degraded to 2-aminomuconic acid or enzymatically converted into the neuroprotective picolinic acid by the 2-amino-3-carboxymuconate-semialdehyde decarboxylase, or non-enzymatically transormed into QUIN (predominantly in dendritic cells infiltrating the CNS and in microglia), a neurotoxic precursor of NAD^+^ and NADP^+^ (Figure 1).

### 2.2. Methoxyndole Pathway

Another direction of TRP metabolism is through the methoxyndole/serotonin pathway. First, TRP is converted to 5-hydroxy tryptophan by the tryptophan-5-hydroxylase, which afterwards is transformed into 5-hydroxytryptamine (also known as serotonin) by the aromatic L-amino acid decarboxylase enzyme. Second, serotonin is acetylated by an alkylamine N-acetyltransferase forming N-acetylserotonin, which in the end is methylated by an acetylserotonin O-methyltransferase to form melatonin (Figure 1).

## 3. Neuroactive Metabolites of Tryptophan

### 3.1. Kynurenic Acid

As mentioned before, KYNA is the end product of one of the three separate branches of the KP. The formation of KYNA is not only segregated from the other pathways (mainly the QUIN branch) enzymatically but physically as well [8,35,36]. In contrast to other products of the KP, KYNA—based on in vitro studies—is mainly considered to be formed in astrocytes, while the other metabolites are produced in microglial cells [37,38,39,40,41,42,43]. Additionally, opposed to several other KP metabolites—because of its polar nature and the lack of transporter mechanisms—KYNA produced in the periphery cannot cross the BBB. In order to exert its function in the CNS it has to be de novo synthesized there, for most of which (~75%) the KAT2 enzyme is responsible [44]. The reported concentrations of KYNA in the brain are 0.2–1.5 μM [45,46,47] (Table 1).

KYNA possesses neuroprotective properties, as it is an endogenous, broad-spectrum competitive antagonist of all three ionotropic glutamate receptors. It has a stronger affinity for NMDARs and weaker antagonistic effect on kainate and AMPA receptors. KYNA exhibits a particularly high affinity for the strychnine-insensitive glycineB binding NR1 site of the NMDA receptor, as it is able to bind to it and block its activity already in the low micromolar concentrations (IC50 ∼ 7.9–15 μM) [69,70,71]. KYNA can bind to the NMDA recognition site of the receptor as well, albeit much higher concentrations are needed for inhibition (IC_50_ ∼ 7.9–15 µM for the NR1 subunit vs. IC_50_ ∼ 200–500 µM for the NR2 glutamate-binding site subunit of NMDAR) [70,72]. In low concentrations, the effect of KYNA on AMPA receptor-mediated responses is paradoxically facilitatory (nanomolar to the micromolar range); neuroinhibition is only achieved at high concentrations (micromolar to the millimolar range) of KYNA [73,74]. The inhibition of the NMDA receptors by KYNA has been shown to be able to defend the neurons against the toxic effects of excessive Ca^2+^ influx—which is one of the most investigated and proven processes in neurodegeneration—via the inhibition of extrasynaptic NMDA receptors during excitotoxic insults.

The peripheral blockade of the KMO enzyme has been shown to increase l-kynurenine levels in the blood, which can readily cross the BBB, and is subsequently transformed into KYNA in the CNS. In the CNS, parallel to the rise of KYNA, a drop was observed in the extracellular glutamate level along with a reduced level of synaptic loss, decreased amount of anxiety-related behavior as well as microglial activation in animal models of Alzheimer’s disease and Huntington’s disease, respectively [75].

In a pivotal study in 2001 it was suggested, that KYNA can noncompetitively bind to the α7 homomeric nicotinic receptors as well [76]. This theory, however, is under heavy dispute nowadays, as to date there is more evidence in support of the fact that KYNA does not influence nicotinic Ach receptors (for a very detailed review on the topic see [73,77,78]). The effects previously attributed to the blockade of the α7 homomeric nicotinic receptors might in fact be due to KYNA’s well established and proven actions on other receptors, which are concomitantly expressed on neurons producing α7 homomeric nicotinic receptors. 

KYNA is also an endogenous, but—compared to other endogenous ligands—weak activator of the G-protein coupled receptor GPR35 [48,79,80,81]. GPR35 was discovered at the end of the last century, designated an orphan receptor for almost two decades, thought to be expressed predominantly in the gut and immune cells. Recent studies have shown, however, that GPR35 is in fact the receptor for the mucosal chemokine CXCL17. It is expressed in mucosal tissue, monocytoid cell lines, CD14^+^ monocytes, T cells, neutrophils, dendritic cells, and in lower levels in B cells, eosinophils, basophils, and on iNKT cells [48,82,83,84]. In the CNS, GPR35 has been found on astrocytes, and has also been linked to nociception and neurotransmission in the CA1 region of the hippocampus [85,86]. The extent to which the agonistic activity of KYNA is relevant physiologically or under the several pathological conditions that GPR35 has been linked to, unfortunately remains unclear thus far [80].

Furthermore, KYNA acts as a potent endogenous agonist at the aryl hydrocarbon receptor (AHR), a transcription factor in the helix-loop-helix (bHLH) Per/ARNT/Sim family, which is expressed in tumor cells, as well as various cells of the immune system [54,87]. In recent years, it has been shown that not only KYNA, but also other tryptophan metabolites (KYN, NAS), are able to activate AHR responses resulting in different downstream effects [55,88]. The binding of kynurenines to AHR results in the internalization of the receptor into the nucleus where it binds to target genes and activates their transcription [89]. Previously AHR was considered a xenobiotic-sensing receptor that is involved in the metabolism of exogenous toxins. More recent data suggests, however, that it has a function in immune-regulation, carcinogenesis, and tumor growth (through the promotion of the generation of immunosuppressive T cells that support cancer development). It has also been shown to play a role in modulating the synthesis of inflammatory mediators [54,90,91]. The exact role of the AHR in the pathways that it is involved in, however, is yet to be clarified in detail.

Additional to its actions at various receptors, an increasing amount of data shows that KYNA also exhibits potent antioxidant traits in the CNS at physiological conditions [67]. In vivo and in vitro data evidence suggests that KYNA is not only able to halt lipid peroxidation, but can act as a scavenger for hydroxyl and superoxide anions as well as other free radicals, further expanding its diverse neuroprotective properties [92,93].

### 3.2. N-Acetylserotonin

An intermediate product of the MP of the TRP metabolism, N-acetylserotonin (NAS) has been shown to possess potent antioxidant, antiischemic and antidepressant properties in mice, and was also able to mitigate the neuroinflammation in mice with experimental autoimmune encephalitis (EAE), the most ubiquitously used model of MS [94,95,96]. As expected, high levels of NAS were isolated from the CNS of mice (from the cervical lymph nodes), especially in the recovery phase of EAE. In fact, NAS has been shown to easily cross the BBB, where it elicited virtually no toxicity. Even though there is an increasing amount of evidence for the presence of the enzyme-producing NAS in organs with function related to the immune system (spleen, bone marrow, and thymus), the exact source of NAS on the periphery remains yet to be pinpointed [97,98].

Recent evidence also suggests that IDO-1 and AHR are functionally intertwined in the modulation of immune responses through a positive feedback loop involving the kynurenine pathway [99]. L-kynurenine is a major endogenous activating ligand of the AHR. The activation of AHR by KYN increases both the expression and the activity of IDO1 in conventional (CD11c+) dendritic cells (where IDO-1 has the highest expression and catalytic activity). This in turn results in the upregulation of TRP breakdown and increased amounts of KYN, which further promotes AHR activity, thus creating a positive feedback loop [15,16,55,88].

A recent study explored the immunomodulatory effect of NAS in EAE. NAS was able to ameliorate the inflammation in EAE in mice with functioning IDO-1 and AHR genes. This effect was lost, however in IDO-1^−/−^ and/or AHR^−/−^ knockout mice [97]. NAS conferred an immunosuppressive effect on dendritic cells via positive allosteric modulation of the IDO1 enzyme. After binding to an allosteric site on the AHR increased IDO-1’s catalytic activity, but no changes in gene or protein expression levels were seen. This resulted in increased AHR activation and immunosuppression via the aforementioned feedback loop [97,100].

Not so long ago, decreased IDO-1 expression and KYN levels (but not TDO expression) were found in the peripheral blood monocytes (PBMC) of relapsing-remitting MS (RRMS) patients compared to healthy controls [23]. When stimulated with IFN-γ, a significant increase in kynurenine production and IDO-1 activity was seen in PBMCs from healthy subjects but not RRMS patients. However, the deficient IDO-1 activity seen in MS patients’ PBMCs could be increased to levels comparable to those of healthy controls after incubation with NAS.

Up to the present day, the main therapeutic approach in the treatment of MS always has been relatively unselective immunosuppression with drugs that impair the adaptive immune response and the activation and proliferation of T and/or B lymphocytes. The deficiency of IDO-1 enzyme has been reported in a number of autoimmune disorders. Emerging evidence suggests that IDO-1 is a novel type of immune checkpoint molecule—a family of molecular regulators that are pivotal parts of the immune system to obtain self-tolerance and to prevent the development of autoimmune conditions—with diverse effects on both the effector and regulatory arms of the human immune response [101,102,103]. The discovery of an endogenous, IDO-1-selective positive allosteric modulator, that has the potency to restore the activity of the deficient enzyme to the physiological level may pave the way for the development of novel drugs for MS with a completely different mechanism of action than before.

### 3.3. Quinolinic Acid

The increased level of quinolinic acid (QUIN) in the CNS has been suggested to be a crucial element in the pathogenesis of several neurological diseases (including, but not limited to, Huntington’s disease, Alzheimer’s disease, schizophrenia, autism, depression, and epilepsy) [104]. The neurotoxic properties of QUIN are manifold, well-described, and have been investigated in depth in the past decades. QUIN’s neuroexcitatory properties can be attributed to several mechanisms, but are mainly due to an extremely specific, though weak (ED50: >100 μM) competitive agonism of the NMDA receptor containing the subunits NR2A, NR2B, and NR2C [5,105].

The NMDA receptors are unequivalently sensitive to QUIN based on their subunit composition, thus QUIN exerts different levels of toxicity in different sites of the brain (because of the different subunit composition of the receptor in different localisations), based on the dominant receptor type in a given anatomical region. It is more than 10-fold more preferential for the NR2B subunit of the NMDA receptors, which are mainly found in the forebrain (abundant in the neocortex and the hippocampus) than it is for the NR2C hindbrain-specific subunit (located mostly in the hindbrain, particularly in the cerebellum and the spinal cord) [106,107,108,109,110,111]. The undisputable, obligatory role of NMDA receptor activation in QUIN mediated neurotoxicity is supported by the findings that all of its toxic effects studied to date can readily be prevented by NMDA receptor antagonists [1,112]. QUIN can also cause excitotoxicity via the stimulation of synaptosomal glutamate release, inhibition of glutamate reuptake by astrocytes, and the enhancement of reactive oxygen species formation. Reactive oxygen species formation by QUIN requires the presence of Fe^2+^ and the subsequent autooxidation of Fe^2+^-QUIN complexes, which process can be negated by iron chelation [113]. QUIN also enhances lipid peroxidation for which the presence of Fe^2+^ ions is also obligatory. On the other hand, the presence of FeCl2 deteriorates QUIN’s ability to activate the NMDA receptors [114,115,116]. QUIN is also able to cause damage by the depletion of various endogenous antioxidants and phosphorylate certain proteins implicated in various neurodegenerative disorders [5,117,118].

Furthermore, vast amounts of evidence suggest that QUIN does not simply possess neurotoxic effects, but is tightly involved with the immune system. Certain pro-inflammatory cytokines (TNFα, interleukin-1β) promote the production and the toxicity of QUIN, respectively [119,120]. Certain anti-inflammatory cytokines (such as IL-4), however, can diminish the production of QUIN by inhibiting the IDO and TDO enzymes, while the blockade of certain receptors (adenosine A2A for example) can protect neurons from the toxic effects of QUIN [120,121].

## 4. The Kynurenine System and Immunoregulation

Various metabolites of the KP have been shown to have profound effects on the functionality of the immune system. Kynurenines have endogenous immunosuppressive attributes through several complex pathways. They modulate the proliferation and function of several immune cells, while, they in turn, are modulated by the cytokines produced by them. In the next couple of paragraphs, we try to give insight into this circle of effects.

### 4.1. Effects on the Immune Cells

It seems that the kynurenines play a central role in T cell mediated immune responses. TRP metabolites, l-kynurenine in particular, can block antigen-specific T cell proliferation and can even induce apoptosis in these cells [84]. Kynurenines mainly induce negative feed-back loops and cell death in the T-helper-1 (Th1) cell population, while they promote upregulation in the Th2 population. This leads to a relative shift towards Th2 in the Th1–Th2 ratio; thus, kynurenines promote anti-inflammatory responses [84,122].

Their direct effects on the T-helper cells are, however, not the only way through which kynurenines can modulate T cell mediated immune responses. It seems that IDO-1 expression in dendritic cells (DC) induces the generation of a specific subset of T-regulatory (Treg) cells, the FoxP3^+^ cells [123,124]. The main function of these Treg cells is seemingly to inhibit both the Th1 and Th2 cells and “guide” the immune system back to balance [84].

Kynurenines do not exert their immunomodulatory effects solely through the T cells, but also affect other immune cells. Natural killer (NK) cells are essential effector cells of the innate immune system. Kynurenines have been proven to suppress both the proliferation and the function of these cells. Through the generation of reactive oxygen species kynurenines halt their proliferation, and via the suppression of specific triggering receptors responsible for inducting NK cell-mediated killing function, they impair cell function [125,126]. These effects can lead to serious malfunction of these cells. Some data also suggest that the inhibition of IDO-1 in polymorphonuclear cells (PMN) impair their antifungal capabilities [127].

### 4.2. The Effect of Cytokines on Kynurenines

Cytokines, modulatory molecules produced (mostly) by immune cells can be divided into pro-inflammatory (e.g., TNF-α, IFN-γ, IL-1, and IL-6) and anti-inflammatory (e.g., IL-4, IL-10) groups. As the names suggest, their effect on the immune response is in direct contrast to each other. The delicate balance of the amount and function of these molecules is essential to the normal functioning of the immune system. It seems that the expression of pro-inflammatory cytokines stimulate the activity of the IDO-1 enzyme; therefore, they increase the rate of kynurenine production [84].

IFN-γ is the most important cytokine in the induction of the IDO-1 enzyme through transcriptomic regulation of the IDO gene [128]. IL-1 and TNF-α act in synergy with interferons in upregulating IDO activity [129]. Thus, the appearance of these cytokines lead indirectly to the increased production of kynurenines. TGF-β is another very potent IDO-1 inductor mainly in Treg and DC cells [130]. IL-23 levels also correlated with kynurenine production in Huntington’s disease [131]. On the other hand, IL-4 and IL-13 are strong inhibitors of IFN-γ induced IDO-1 mRNA expression and kynurenine production [132].

### 4.3. Gut-Microbiome and Kynurenines

In recent years, a plethora of new information has surfaced about the gut microbiome after the discovery that it not only plays a major role in maintaining a healthy state for the host, but its perturbation has been linked to a vast amount of neurodegenerative and immunological diseases [133,134,135,136,137,138,139].

Not only are the thousands of bacteria colonizing the gastrointestinal tract in constant flux in response to the changes in the nutrition and diet and drug consumption of the host, but the metabolism of these bacteria and the cytokines and molecules produced by them are in a perpetual shift as well [137,140,141]. As mentioned above, TRP is an essential amino acid for humans, but many bacteria found in the gut can synthesize it. Additionally, bacteria are capable of producing neuroactive kynurenines—which can penetrate the BBB—by various other mechanisms [142,143,144,145,146]. QUIN can be de novo synthesized by gut bacteria from iminoaspartate or metabolized from anthranilic acid, but can also be produced non-enzymatically [147,148,149].

Numerous studies investigated the change in the composition of MS patients’ gut bacteria. Changes in several species were detected; some were depleted, while other species were enriched compared to healthy individuals. The exact role, however, the gut bacteria (which have been associated with the development of MS) play in the regulation of the formation of neuroactive kynurenines or how they shift the balance between the neuroprotective KYNA and neurotoxic QUIN, remains unclear thus far [150,151,152]. Furthermore, how exactly the microbially regulated TRP metabolism and KP metabolites synthesized in the gut influence the function and dysfunction in the CNS remains elusive to date. How and where the pathway can be targeted is also of great interest and may hold significant therapeutic potential; however, this is yet to be discovered [153,154].

### 4.4. Disturbances of the Kynurenine System in Neuro-Immunological Conditions

IDO-1 overexpression and tryptophan metabolite disturbances have been found in a number of inflammatory conditions that can affect the CNS. Disturbances in the KP was proven in several acute exogenic inflammatory processes of bacterial or viral origin, as well as in autoimmune conditions such as systemic lupus erythematosus (SLE) and Sjögren’s syndrome.

A recent study found major induction of the KP in bacterial and viral CNS infections that correlates strongly with blood-cerebrospinal fluid-barrier dysfunction and the standard measures of neuroinflammation in the cerebrospinal fluid (CSF) [155]. The most prominent finding was in viral meningitis, with the kynurenine/TRP ratio being the most reliable marker [155].

SLE is a systemic autoimmune condition that can affect several organ systems. Classical signs can be seen on the skin (e.g., “butterfly rash”), some forms of the disease are limited to the skin alone. More often than not, however, the disease affects other organ systems (most frequently the kidneys and the respiratory tract), and in a high number of cases, the nervous system as well. Some studies showed, that up to 75% of patients with SLE show some degree of involvement of the CNS with diverse symptoms both in appearance and severity [156]. Kynurenines have been studied particularly in this type of the disease, labeled as neuropsychiatric SLE. Higher levels of QUIN were measured in the plasma and the CSF of SLE patients compared to healthy controls along with lower levels of TRP [157]. Additionally, significant correlation was shown between the kynurenine/TRP ratio and TNF-α levels, demonstrating a connection between the pro-inflammatory pathway and kynurenines [157].

Sjögren’s syndrome is classically defined as an autoimmune exocrinopathy: the main targets of the immune response are the salivary and lacrimal glands [158]. However, the involvement of the nervous system (either the periphery or the central) can be present in up to 60–80% of patients, ranging from peripheral neuropathy, to MS-like (“MS-mimic”) conditions or neuromyelitis optica spectrum disorder (NMOSD) [159,160]. Disturbance of the kynurenines—not surprisingly—have been found in these patients as well. Analysis of the peripheral blood with flow cytometry of Sjögren’s syndrome patients detected higher expression of IDO-1 in the patients’ dendritic cells as compared to the dendritic cells of healthy controls [161]. Additionally, overexpression of IDO was demonstrated in both T cells and antigen-presenting cells (APC) of Sjögren’s syndrome patients, compared to healthy controls [161,162].

### 4.5. The Role of the Kynurenine System in Multiple Sclerosis

Multiple sclerosis (MS) is an autoimmune, demyelinating, and neurodegenerative disease of the CNS. As such, kynurenines have been investigated in the pathomechanism of the disease in both pre-clinical and clinical assessments and their important role has been suggested by several studies.

#### 4.5.1. Kynurenines and Animal Models of MS

In spite of vigorous and large-scale research conducted during the past decades into the pathogenesis, causes setting up and initiating MS, the exact trigger(s) remain elusive. Taking into account the complexity of the disease, the fact that the currently available and efficient treatments for MS have different targets (some have an effect on T cells, while some target B cell lines), the notion was raised that potentially different pathomechanisms may be underlying the disease recognized as MS. More recently, it was suggested that in fact not a single, but several intertwining pathological processes may be responsible for the demyelination of neurons and formation of lesions. This results in subsequent tissue injury, the cessation of saltatory nerve conduction, and increased axonal vulnerability followed by an eventual loss of neuronal function and successive cell death.

One hypothesis considers autoreactive T cells and macrophages that infiltrate the CNS and attack oligodendrocytes, which myelinate axons, to be the initiators of acute MS lesions [163]. This theory is supported by the oldest and most frequently used model system for studying MS: the experimental autoimmune encephalomyelitis (EAE) model, which is histologically similar to human MS. In its classic form, EAE mimics the chronic form of MS. In this version, EAE is a mainly cell-mediated condition, in which (most commonly) C57BL/6 mice are immunized agains the myelin sheath with peptides of myelin oligodendrocyte glycoprotein and/or lipopolysaccharides along with an adjuvant (most frequently Complete Freund’s Adjuvant, pertussis toxin, or both). The immunization causes autoreactive Th-1 and Th-17 cells [164] to cross the blood-brain barrier which, after a roughly 2-week long incubation period results in inflammation and demyelination, followed by oligodendrocyte and neuronal death that is similar to that seen in MS. The same histopathological results can seen after the adoptive transfer of autoreactive T cells into the CNS [165]. Another frequently used strain of mice (SJL/J) develops a relapsing-remitting form of the disease—the most commonly seen version of MS in humans—after immunization with either intact or fragmented myelin basic protein or proteolipid protein with adjuvants. In this strain the first signs of the disease begin to show 7 to 14 days post-induction. After the initial inflammatory attack subsides, the animals go into remission, after which a remissive phase very similarly to human RRMS subsequent episodes of inflammation, demyelination, and axonal loss occurs [166,167,168].

A different hypothesis, on the other hand, suggests that the first step in the development of new lesions is in fact oligodendrocyte apoptosis and microglial activation in the presence of only a few or no lymphocytes of phagocytes. This culprit event is in turn followed by primary demyelination and a successive, secondary auto-immune inflammatory process, which leads to subsequent lesion expansion and further oligodendrocyte loss [169]. These histological findings are not present in EAE models, which indeed raises the possibility that multiple processes are responsible for new lesion formation in MS. A most recent study suggests that a specific subset of autoreactive T cells producing IFN-γ and, to a lesser extent, IL-17 targeting the β-synuclein protein (which is abundantly expressed in grey matter, where high density of neuronal processes and synapses are present) may at least in part be responsible for the initiation of grey matter lesion formation, expansion, and subsequent gliosis. They were found to be mainly increased in patients with chronic-progressive MS, whereas myelin-reactive T cells (thought to be mainly responsible for white matter lesion formation) were predominant in patients with relapsing-remitting MS. The recent recognition of this cell type argues for the fact that one of the long speculated alternative pathological mechanisms (such as hypoxia or currently undefined soluble toxic factors) that spark the degenerative grey matter process in MS may be, in fact a previously undiscovered subpopulation of autoreactive T cells [170].

In addition to the aforementioned mechanisms, mitochondrial damage with consequent energy failure was found to be a key component in several aspects that constitute to MS pathogenesis and lesion formation [171,172,173,174,175]. The presence of mitochondrial dysfunction has been linked to oligodendrocyte apoptosis and subsequent demyelination [176], the halting of the differentiation of oligodendrocyte progenitor cells [177], the loss of small-diameter axons and lesion progression [178,179], and astrocytic dysfunction [180].

Several disturbances in the kynurenine system have been observed in the EAE model. Additionally IDO-1 and various KP metabolites have been shown to ameliorate autoimmunity and to promote immune tolerance.

This immunomodulating effect of the KP may be in part responsible for the periodic remissions seen in MS and EAE. Increased kynurenine/tryptophan ratios and microglial/macrophage-derived IDO-1 activity have been shown to be present in the brain and spinal cord of mice at the onset of EAE symptoms; meanwhile, a simultaneous fall in IFN-γ levels was observed [181,182]. This suggests the suppression of IFN-γ producing Th1 cells by increased IDO activity. Encephalitogenic Th1 cells secreting IFN-γ have been shown to induce local IDO expression, which in turn suppresses Th1 and Th17 cells and promotes the expansion of immunoregulatory T cell lines (Th2, Treg cells), terminating its own production, therefore, creating a negative feedback loop. This regulatory mechanism may be at least partly responsible for the cyclicality of relapses and remissions seen in EAE. Furthermore, the decreased activity of IDO-1 in EAE, either by genetic deficiency or pharmacological blockage (by the administration of 1-methyl-tryptophan) has been shown to lead to increased Th1 and Th17 responses with decreased Treg responses resulting in faster disease development, a more severe clinical course, and higher amount of spinal cord inflammation, which argues for this hypothesis [181,182,183].

The upregulation of IDO-1, successive tryptophane starvation, and the accumulation of its downstream tryptophan metabolite, 3-HAA, on the other hand, had ameliorated the inflammation. The increased amounts of 3-HAA have been shown to directly suppress the activity of Th1 and Th17 cells, and also to reduce their activity indirectly by increasing the amount of TGF-ß secreted by DCs, which subsequently resulted in the increased generation of Treg cells from naive CD4^+^ cells [181,183]. The impaired immunosuppressive activity of Treg cells on Th1 and Th17 cells and the disruption of regulation between B and T cells, dendritic cells, and natural killer cells have been demonstrated to play a role in the breakdown of self-tolerance and the pathogenesis of MS [184]. Similar results were seen with cinnabarinic acid, a less well-studied endogenous kynurenine metabolite. Systemic administration of cinnabarinic acid was capable of enhancing Treg response at the expense of Th17, proved to be highly protective against EAE. Exogenous cinnabarinic acid was found to enhance endogenous cinnabarinic acid formation in lymphocytes, suggesting the occurrence of a positive feedback loop sustaining immune tolerance [185]. In line with these findings, the protective role of IDO-1 activation in EAE has been demonstrated in another study. Estrogen administration resulted in induced IDO-1 expression in dendritic cells, which in turn has led to concomitant T cell apoptosis and the attenuation of EAE symptoms. This mechanism has been proposed to explain estrogen-mediated EAE suppression and to at least in part underlie the decreased rate of relapses seen in MS patients during pregnancy [186].

Other downstream metabolites of the KP have also been found to have a attenuating effect on EAE symptoms. Both endogenous (3-HAA, 3-KYNA) and orally active synthetic metabolites (N-[3,4-dimethoxycinnamoyl] anthranilic acid) have been shown to suppress the expansion of myelin-specific Th1 and Th17 T cells, inhibit the production of Th1 cytokines and elevate Treg response and ameliorate symptoms in EAE mice demonstrating a pivotal role of downstream kynurenine metabolites in IDO-1 mediated EAE suppression [183,187].

In summary, various metabolites of the KP may suppress auto-immunity and ameliorate symptoms in EAE not only through local tryptophan depletion, but also through influencing T cell differentiation (promoting the expansion of regulatory T cells, while limiting the expansion of autoreactive T cells) mediated through cytokines derived from both T cells and dendritic cells.

In addition to the upregulation of IDO-1, the significantly increased activity of KMO was also observed in the spinal cord of rats with EAE. As a consequence of this increase in KMO activity, neurotoxic levels of QUIN and 3-HK were measured. The addition of a selective KMO inhibitor (Ro 61-8048) resulted in a robust reduction of QUIN and 3-HK levels with a concomitant rise in KYNA concentration. Interestingly, however, this change of balance between neurotoxic and neuroprotective kynurenines did not influence the outcome and severity of EAE. This points against previous findings and suggests that neurotoxicity mediated by QUIN and neuroprotection conveyed by KYNA do not have a vital role in the outcome of EAE [181,188].

Another pathological aspect in EAE was the translocation of the KMO enzyme. In healthy controls, KMO immunoreactivity has been reported to be present in the cytoplasm of both neurons and astroglial cells (most likely inside the mitochondria of these cells). In the case of rats with EAE, however, a very intense KMO immunoreactivity was seen in subependymal, subpial, and perivascular locations in cells that expressed both the inducible nitric oxide synthase enzyme and class II major histocompatibility complex, suggesting these cells to be macrophages. These findings support the notion that the cells of the immune system are responsible for the inflammation are also the source of neurotoxic kynurenines in the CNS of rats with EAE [181,188]. Under pathological conditions (such as in MS and EAE), the breakdown of the blood-brain barrier allows for uncontrolled leukocyte infiltration. Thus, a substantial part of the elevated amount of QUIN measured in the CNS may actually be derived from macrophages originating from the periphery. The release of cytotoxic, pro-inflammatory cytokines by macrophages and microglia concomitant to increased QUIN production by macrophages leads to an amplifying feedback mechanism that further stimulates QUIN synthesis and likely contributes to MS lesion pathology and expansion.

In addition to its elevated levels, the oligodendrocyte apoptosis-inducing properties of QUIN were also observed in the spinal cord of rats with EAE [189,190]. Chronic, low dose exposure to QUIN has been shown, however, to be toxic and cause the destruction of not only oligodendrocytes, but astroglial and neuronal cells as well [190,191,192]. In addition to the previously mentioned ways, QUIN seems to play a role in neurodegeneration through changing the structure of several important proteins [193], which reduces the cells’ ability to neutralize reactive oxygen and nitrogen species and free radicals [194,195], affects the glutathione redox potential [196], depletes superoxide dismutase activity [197], enhances lipid peroxidation [198,199] and disrupts mitochondrial function [200,201].

It is of utmost importance to mention a notable pitfall of EAE. As mentioned previously, in the induction of the disease, the animals are not only immunized against various elements constituting the myelin sheath, but mycobacterium tuberculosis is also a component of the Freund’s adjuvant used in the process of auto-immunization in some models. It was demonstrated that the increased levels of QUIN and 3-hydroxy-kynureninase levels observed in the spinal cord of rats with EAE [189] were the consequence of higher IDO-1 and KMO expression levels and activity [188]. The increased activity of IDO-1 and KMO is, however, likely the result of an immune reaction to the presence of bacterial antigens but not myelin proteins. This allegation is supported by the fact that in one study, solely mycobacterium tuberculosis, but not MBP, was found to be a potent activator of IDO-1 [202]. Furthermore, increased IDO-1 activity and significantly lower tryptophan concentrations were found in patients with pulmonary tuberculosis compared to healthy controls [203]. Increased IDO-1 activity was also associated with poor outcomes in patients with bacteremia and cancers [204]. This further underlines the KP’s role in xenobiotic sensing immunological processes.

#### 4.5.2. Kynurenine Metabolite Changes in MS

Not only pre-clinical data are available on the kynurenines’ role in MS, but a number of studies supplied data from humans as well. In addition to the precise pathomechanism of MS being unknown yet, the exact role the KP plays in it is unclear as well. Therefore, it is not surprising that conflicting data are available regarding the various changes observed in the KP and its metabolites in MS. There is an agreement, however, that a significant dysregulation of the KP is present in MS. Some evidence suggests, that the induction of the kynurenine pathway is mediated by pro-inflammatory cytokine-cascades, as described above [193,205,206]. Many of the previously described proinflammatory factors and cytokines modulating the KP are known to be altered in MS as well. Therefore, it is rational to assume that changes in the KP will be present in patients with MS who show disease activity, e.g., at times of acute lesions formation/expansion, when increased inflammation in the CNS is present. It is also logical to presume that during chronic stages of the disease when little or no CNS inflammation is present only minor or no changes are expected to be seen in the KP. Activation of the kynurenine pathway results in two very distinct and opposite events. Short-term benfits of the KP’s activation arise in the form of decreased T cell proliferation (via the previously discussed pathways and feedback loops), leading to immunosuppression, while chronic activation of the KP enzymes induces the production of neurotoxic metabolites and plays a role in preventing the innate repair mechanism of remyelination [207].

The first report exploring the connection between MS and the KP is from 1979, the study has found decreased levels of tryptophan in the serum and CSF of MS patients compared to controls [208]. Later studies in the 1990s, however, have reported controversial results about CSF and serum tryptophan levels in MS [209,210]. Additionall, a negative correlation was found between CSF levels of tryptophan and neopterin during acute relapse, possibly representing IDO-1 activation in CNS-infiltrating macrophages [209]. Following these studies, another group failed, however, to detect a significant baseline difference in the plasma L-kynurenine/tryptophan ratio between relapsing-remitting MS and samples from healthy controls. Interestingly, however, an increased L-kynurenine/tryptophan ratio was detected after treatment with INF-β, implicating IDO-1 activation as a potential mode of action of INF-β products, which were widely used at the time as the first-line treatment of MS (for more details, see the chapter on treatment effects) [211]. These results fall in line with the findings of Rothammer et al., who detected a global decrease of circulating AHR agonists in relapsing-remitting MS patients compared to healthy controls. They have also reported increased global AHR activity during relapse and diminished AHR activity (reflecting decreased AHR agonist levels) in remission in the serum of MS patients implicating the role of the endogenous AHR agonist L-kynurenine. Moreover, AHR ligand levels in patients with mild clinical impairment despite a longstanding disease were unaltered as compared to healthy controls [212].

Several studies since then have succeeded in confirming numerous alterations in the KP in different MS subtypes at various points of the disease, which are in support of the aforementioned ideas. KYNA levels were found to be decreased during the remissive phase and elevated during acute clinical exacerbation in the CSF of RRMS patients compared to healthy controls [7,213,214]. Elevated levels of QUIN were associated with oligodendrocyte, astrocyte, and neuronal loss, while decreased amounts neuroprotective metabolites such as kynurenic acid and picolinic acid were also reported in MS patients [8]. Another study showed that pathological amounts of QUIN might be responsible for the abnormal tau-phosphorylation seen in the progressive phase of the disease [215]. Lower levels of both KAT1 and KAT2 enzymes were also found in MS patients’ brain tissue by histopathological processing [216].

A recent study investigated the KP metabolomic profile of MS patients and the balance between its two metabolites, KYNA and QUIN. The ratio of these two metabolites is pivotal, as it determines the overall excitotoxic activity the KP has at the NMDA receptor. Based on the metabolomic analysis and profiling of the KP from the serum of MS, Lim et al. have built a predictive model for the disease subtypes using six predictors. The model evaluated the levels of KYNA, QUIN, tryptophan, picolinic acid, fibroblast growth-factor, and TNF-α (in order of relevance) to predict the disease course with up to 85–91% sensitivity [217]. This points toward the notion that the metabolic profiling of the KP may become a potentially useful biomarker in the future, mainly in separating the different clinical courses of the disease early after disease onset. In the same study KYNA levels were reported to be the highest in the relapsing-remitting group compared to both healthy controls and patients with a progressive disease type. The lowest levels of KYNA were measured in primary/secondary progressive MS patients [217].

Another study investigated the potential difference in IDO-1 expression in and activity in peripheral blood mononuclear cells between healthy controls and RRMS patients in remission and in the acute phase, and whether a change in IDO-1 activity/expression was indicative of an onset of a relapse. IDO-1 expression and activity remained unchanged between healthy controls and patients in the acute phase and between healthy controls and stable RRMS patients. The activity of IDO-1 was shown to be independent of the onset of a relapse. Increased IDO-1 expression and decreased levels of IFN-γ were seen, however, in MS patients with a relapse before corticosteroid treatment compared to patients in remission. Glucocorticoid-induced disease remission resulted in a significant reduction of IDO-1 and IFN-γ gene expression, IDO-1 catalytic activity. Serum neopterin (a protein biomarker for inflammation released by macrophages upon IFN-γ stimulation) concentrations followed the same trend as IDO-1 expression and activity. The pitfalls of this study were the relatively low amount of subjects (15 healthy controls, 21 patients in the acute phase, and 15 in remission), and the fact that different patients were used in the remissive and active groups. The same patient was not examined in both the acute and in remissive phase; therefore, intraindividual changes in IDO-1 activity and its role as a potential relapse indicator could not have been established [218].

A recent study by Rajda et al. investigated the connection and association between biomarkers of inflammation (neopterin), neurodegeneration [neurofilament light chain (NFLc)], tryptophan, and kynurenine metabolites measured at diagnosis in the CSF of MS patients (32 RRMS and five CIS patients) and healthy controls (*n* = 22). Compared to controls, all of the measured markers were elevated in the CSF of MS patients, except for KYNA, which showed no change in MS patients compared to controls. Additionally, a strong positive correlation was found between NFLc normalized for age, neopterin, and QUIN [213,214].

Similarly to the study reported by Rajda et al. [207], Aeinehband et al. [219] failed to show a difference in the levels of KP metabolites (tryptophan, kynurenine, KYNA, and QUIN) in the CSF of MS patients and control subjects with non-inflammatory or inflammatory neurological diseases, when the MS patients were pooled. The study included 71 MS patients, 20 non-inflammatory neurological disease control patients, and 13 control patients with inflammatory neurological disease. After the MS patients were stratified according to their disease subtypes and different phases of the disease significant differences in KP metabolites could be demonstrated. Increased QUIN concentrations and quinolinic acid/kynurenine ratios were seen in RRMS patients during the relapsing phase, whereas patients with secondary progressive MS had lower tryptophan and KYNA levels. Patients with a primary progressive disease similarly to control patients with an inflammatory neurological disease showed increased levels of all evaluated tryptophan metabolites. This further strengthens the findings of Lim et al., in that clinical course and disease activity are reflected by changes in KP metabolites. These findings also raise the possibility that someday clinical course and disease severity may be predicted by profiling the KP’s metabolites [219].

#### 4.5.3. The Kynurenine System and Depression in MS

Psychiatric disorders, especially depression is, however, one of the most frequent comorbidity with MS, its prevalence can be as high as ~30.5% [220] even among MS patients. Several studies have linked various disturbances in the kynurenine system and depression. Development of depression is associated with decreased serotonin, melatonin, and N-acetyl-serotonin levels, which in turn have been linked to increased immuno-inflammatory pathway activity in patients with depression [221]. Not so long ago, changes in serotonin transporter levels in MS patients were confirmed. This suggests that alterations in serotonin availability and subsequent disturbances in N-acetyl-serotonin and melatonin production are likely to occur in MS patients in a similar manner to that seen in patients with depression. It was hypothesized by some authors, based on the disease modulating and remyelinating effect of melatonin, that depression may not be in fact a frequently occurring comorbidity, but rather a symptom of MS, a part of the disease itself [222,223]. The levels of several inflammatory cytokines (such as IL-1 β, IL-6, IL-18, TNF-α, and IFN-γ) have been shown to be altered in MS. Many of the same cytokines (especially IFN-γ) also increase IDO-1 activity and expression, and therefore, can cause subsequent depletion of serotonin, N-acetyl-serotonin, and melatonin [224]. Kynurenine is able to cross the blood-brain barrier and can increase tryptophan catabolite levels in the CNS, which is another proposed mechanism via the KP and its metabolites can contribute to the depression, somatization, and fatigue seen in MS [225]. A recent study investigated the correlation between alteration in KP metabolite levels in the CSF of MS patients with short disease duration and an active disease and the presence of neuropsychiatric symptoms. Depressed MS patients were demonstrated to have higher KYNA/tryptophan and kynurenine/tryptophan ratios, which was mainly due to low tryptophan levels.

#### 4.5.4. Treatment Effect on the Kynurenine System

Even though a plethora of disease-modifying treatments are available for the treatment of MS nowadays, the most experience and data exists for one oldest approved drugs, interferon-β-1b (IFN-β). Beta interferon is a standard first line treatment, two shorter (BENEFIT and BEYOND), and one very long term phase IV study (LTF) has provided safety and efficacy data on the effects of IFN-β-1b treatment of MS patients. Amirkhani et al. have found increased plasma and serum concentrations of L-kynurenine and increased kynurenine/tryptophan ratios in patients after treatment with IFN-β compared to baseline [211,226]. In contrast, lower levels of kynurenine and N’-formylkynurenine were detected in the serum of patients receiving IFN-β treatment compared to controls in another study [227]. Depression was one of the most common adverse events reported in patients treated with IFN-β [228,229,230]. Treatment with interferons in other diseases has been known to increase the risk of depression. One of the most potent inducers of IDO-1 (in addition to the previously mentioned INF-γ) is IFN-α, which is used in the treatment of chronic hepatitis C. Several earlier studies reported a decrease in blood tryptophan concentrations and a concomitant increase of KYN which has been linked to IFN-α induced depression, suggesting the role of IDO-1 in the process [231,232,233,234]. This raised the question of whether IFN-β treatment was at least in part responsible for the increased prevalence of depression in MS patients. In vitro studies conducted with human monocyte-derived macrophages support this observation. IFN-β has been shown to be able to induce QUIN production and enhance IDO expression in macrophages in therapeutic doses used in MS [235]. However, it remains unclear whether the changes seen in KP metabolite levels mediated by IFN-β are indeed causatively involved in the development of depressive symptoms in interferon treated MS patients. It is also undetermined whether IFN-β-mediated IDO-1 induction is the reason of the low efficacy of IFN-β treatment in improving MS symptoms [8]. The basis for this hypothesis comes from cell culture experiments conducted inhuman macrophages where IFN-β-treatment resulted in increased levels of QUIN production [235], combined with the fact that QUIN is a weak, but well-established NMDAR agonist [8]. Thus far, however there is no direct evidence demonstrating that the use of IFN-β in therapeutic concentrations result in increased CNS QUIN levels in high enough concentrations to be the sole cause of depressive symptoms in MS patients.

In summary, the results of these studies suggest that the KP, most notably the activity of IDO-1, might be downregulated or unaltered in stable MS, probably contributing to disease pathogenesis, whereas its upregulation can be seen during acute inflammatory relapses, most probably reflecting an endogenous counter-regulatory reaction, which responds to anti-inflammatory therapy. A rise in downstream kynurenine metabolism additional to IDO-1 activity can be seen during an acute inflammatory exacerbation in MS. Furthermore the imbalance between neurotoxic and neuroprotective metabolites of the kynurenine pathway favoring the neurotoxic ones might contribute to neurodegeneration in progressive MS subtypes in part via NMDA receptor-mediated excitotoxicity. 

#### 4.5.5. Kynurenic Pathway and Redox Disturbances in Neuroinflammation and Multiple Sclerosis

The de novo, eight-step synthesis of NAD^+^ from TRP takes place in the liver, neuronal, and immune cells, and starts with the conversion of quinolinic to nicotinic acid mononucleotide (NaMN) by QUIN phosphoribosyltransferase in the presence of Mg^2+^ The subsequent step in the metabolism takes place in the nucleus, mitochondria, and the Golgi apparatus by the nicotinamide mononucleotide adenyl transferase enzymes (NMNAT1, 2, and 3). NaMN is converted into desamido-NAD^+^ with the consumption of ATP. The last step in the cascade is the amidation of desamido-NAD^+^ in the presence of glutamine. NAD^+^ can be synthesised by at least three additional, TRP-independent routes called salvage pathways. First, it can be produced from nicotinic acid, which is converted to NAD^+^ via the three-step Preiss-Handler pathway, taking place in the liver, kidney, intestine, and the heart. A second option is by the nicotinamide salvage pathway (called the two-step Nampt pathway, taking place in adipose tissue, liver, kidneys, and immune cells) in which nicotinamide phosphoribosyltransferase (NMAPT, the rate-limiting, glycosyltransferase enzyme of the pathway) converts nicotine amide and 5-phosphoribosyl-1-pyrophosphate to nicotinamide mononucleotide (NMN) and pyrophosphate, respectively. NMN is afterwards transformed to NAD^+^ by the action of NMNAT1, -2, and -3 in the presence of ATP. The third way is the phosphorylation of nicotinamide riboside to NMN by nicotinamide riboside kinases (placed in cardiac and skeletal muscle, neuronal tissue) [236,237,238]. In physiological conditions from all the possible mechanisms to create NAD^+^, the de novo synthesis from TRP seems to be the main source [239]. NAD^+^ is not solely a pivotal cofactor in several biochemical pathways but acts as an electron transporter. It also serves as a substrate for the DNA damage sensor and putative nuclear repair enzyme, poly(ADP-ribose) polymerase (PARP). PARP is a nucleotide polymerase abundant in the nucleus, which (in concert with DNA dependent protein kinases) is responsible for upkeeping the integrity of the DNA double-strand. Excessive oxidative stress can damage the DNA causing double-strand breaks, which is the activating signal for the PARP enzyme. In neurons affected by glutamatergic excitotoxicity an increase in intracellular oxidative stress and PARP activity was observed [240]. The activation of PARP results in the poly-ADP-ribosylation of the enzyme itself and other molecules participating in the attempted repair of the damaged DNA segments. To fuel this machinery, NAD^+^ and NADP^+^ are used up, resulting in the depletion of the NAD^+^ and NADP^+^ stores of the cell and the release of nicotinamide as a by-product. The fall in intracellular NAD^+^ levels following PARP activation has been observed in many cell lines in the CNS in numerous neurodegenerative disorders, neuroinflammation, aging, and infections, and following the exposure to various excitotoxins and free radicals [241,242,243]. Excessive activation of PARP has been shown to result in cell lysis and eventual death via the depletion of not only the intracellular NAD^+^ but the ATP storage as well, causing energy restriction in the cell and a fall in neurotransmitter levels in the brain. In an attempt to recover the used up NAD^+^ from nicotinamide, the aforementioned salvage pathways are activated. To do so, ATP is required, indirectly for the reaction catalyzed by NAMPT and directly for the NMNAT enzyme. The upregulation of the salvage pathways consumes the remaining ATP of the damaged cells. When excessive DNA damage occurs, this becomes a vicious circle, which consumes the NAD^+^ and energy reserves of the cell to the brink of energetic collapse. In these situations, the accumulation of poly(ADP)ribose by PARP can induce apoptosis to ensure the timely death of the cell with severely enough damaged DNA, thus reducing the chance for tumor formation [244]. This mechanism is supported by recent data, where it was shown that the blockade of PARP in resulted in the preservation of both NAD^+^ and ATP levels in cells exposed to oxidant injury, cell lysis was also prevented. DNA damage, however—as expected—was not mitigated, highlighting the crucial role that PARP may play in the pathogenesis of neurodegenerative diseases in which elevated levels of free radicals, excitotoxicity, and pro-oxidants have been confirmed [240].

As mentioned before, to date, only microglia, dendritic cells, astroglia, and macrophages have been shown to express 3-HAO in the CNS, which produces QUIN [245]. IFN-γ has been shown to be the primary activating factor of dendritic and microglial cells, as well as macrophages in both the periphery and in the CNS. Activation by IFN-γ readily modulates these cell line’s metabolism and increases their antimicrobial activity through the upregulation of several pathways. It activates the secretion of complement pathway components, induces the production of reactive oxygen species, and upregulates nitric oxide synthase activity. Additionally, it enhances the expression of MHC antigens and upregulates the secretion of several cytokines (including, but not limited to IL-1β, IL-6, TNF-α, platelet-activating factor, macrophage chemotactic protein, etc.), all of which play key roles in the induction of neuroinflammation [239,246]. The rate-limiting enzymes—IDO1,2—of the kynurenine pathway are also potently induced by IFN-γ, leading to the elevated production of neuroactive kynurenines in the CNS by the cells expressing the enzymes. This results in an increased level of all the end products of the KP as well as picolinic acid. Following IDO activation by IFN-γ induction, in addition to other various effects, increased intracellular NAD^+^ concentrations were measured in astrogliomas, followed by increased TRP catabolism [6,246,247]. How exactly the production of pro-inflammatory and oxidative metabolites vs. anti-inflammatory members of the KP are orchestrated to date is controversial. 3-hydroxyanthranilic acid—a potent antioxidant KP metabolite—inhibits the nitric oxide synthase 2 enzyme in macrophages and can also suppress the inducible nitric oxide synthase, readily inhibiting the NF-κB activation even at sub-millimolar concentrations [248,249]. The increased production of 3-hydroxyanthranilic acid by activated mononuclear phagocytes present in inflamed neuronal tissue may, therefore, mitigate the damage caused by increased oxidative stress and may explain the observed increase in TRP catabolism in these tissues. The increased production of kynurenine and QUIN under these conditions, however, remains controversial [250]. Another kynurenine metabolite, cinnabarinic acid —produced in peripheral mononuclear cells and under oxidative stress via non-enzymatic reactions—has been shown to be a pivotal molecule for immune response. The concomitant expression of IDO and enzymes responsible for the production of free radicals has been demonstrated in immune cells to redirect the kynurenine pathway from the production of quinolinic- or picolinic acid towards cinnabarinic acid. Cinnabaranic acid has been proven to modulate the immune response; recently, it was confirmed to be one of the endogenous ligands of the AHR. Via the stimulation of the AHR, it increases the IL-22 production in human T cells pushing the balance between towards regulatory T cells over IL-17 producing T cells [239,251].

Various alterations in the redox pathways, in the KP, levels of free radicals, and NAD^+^ production and depletion have been observed in vivo (in both animal models of MS and humans). Neurons in EAE have been shown to be extremely vulnerable to the degenerative characteristics of MS under conditions of NAD^+^ deficiency [252]. An increased net level of NAD^+^ was observed in the CNS in the EAE model; however, it is suggested that this was due to the increased NAD^+^ content of the lymphocytes and APCs infiltrating the CNS, meanwhile, neurons were actually in a state of NAD^+^ deficiency [238].

Cumulating evidence suggests that TRP metabolism and professional APCs expressing IDO-1 are key components in MS. Accordingly, serum TRP concentrations were found to be low and linked to poor prognosis in autoimmune disorders involving IDO-1 activation and Th1 type cellular immune response [253]. Despite being a key player in immune regulation and TRP metabolism, IDO-1 seems to be a double-edged sword. On one hand, it exerts therapeutically beneficial effects of decreasing autoreactive T cell proliferation; however, persistent activation of IDO-1 has been shown to lead to NAD^+^ depletion in otherwise healthy neighboring collateral tissues [254]. The suppression of autoreactive T cell proliferation by IDO-1 is in part due to its rapid upregulation in professional APCs—such as microglia, macrophages, and dendritic cells—after being exposed to Th1 type cytokines (most prominently IFN-γ and CD40L, but also TNF-α). The thus induced IDO-1 uses up extracellular TRP, which results in the halting of proliferating T cells at the mid-G1 arrest point. The cells cannot progress further from this point in the absence of another T cell signal, even after the restoration of extracellular TRP levels, which eventually causes autoreactive T cells to diminish [255,256]. One of the most potent modulators of the adaptive immune system, dendritic cells—in which cell line IDO-1 is exceptionally highly expressed—have been linked to relapses/chronicity of neuroinflammation, and to the breakdown of tolerance to CNS autoantigens [257,258]. The importance of immunomodulation exerted by IDO-1 is further underlined by the fact that the ameliorating effect of stem cells in EAE pathogenesis was abrogated by the inhibition of IDO-1. In line with this, other studies not involving stem cells confirmed IDO-1 suppression to exacerbate EAE [181,182,259].

Another way through KYN can exert its immunomodulatory effects is by being a precursor for NAD^+^ production. It is proposed that kynurenine is taken up by APCs with induced IDO-1 activation and is readily metabolized into NAD^+^ to act as a second messenger, which may be more important to kynurenine’s immunomodulatory effects, than the depletion of TRP itself [260,261]. This theory is supported by the confirmed ability of plasmacytoid DCs—a subset of professional APCs—to create a profoundly suppressive microenvironment. This is completely dependent on IDO-1 activity via the constant stimulation of CD4^+^, CD25^+^, and Foxp3^+^ Treg cells, which send tolerogenic signals to other T cells [261]. In light of this information, the production of NAD^+^ and its activity as a second messenger may be just as important in immunosuppression in APCs, as is the actual local depletion of its precursors.

In a mammalian EAE model, the depletion of NAD^+^ has been linked to PARP activation based on immunostaining findings of its metabolite poly(ADP-ribose). It was abundantly found not only in astrocytes surrounding demyelinated EAE plaques but in microglia, oligodendrocytes, and endothelial cells surrounded by microglia and infiltrating peripheral blood cells as well [262].

TRP levels have been shown to be decreased both in the serum and CSF in MS patients (see before) [8,193,209,263]. In RRMS patients, a more than 50% reduction in serum NAD^+^ levels, as well as a two-fold increase in NADH levels and three-fold reduction in the NAD^+^/NADH ratio was seen compared to controls. Furthermore, among MS patients NAD^+^ levels were the highest among RRMS patients, followed by primary progressive MS patients, and were the lowest in secondary progressive MS [264]. Administration of pharmacological doses of nicotinamide and calorie restriction was, however, able to rise the NAD^+^ levels and ameliorate EAE pathogenesis in animal models [265,266,267,268].

In summary, during neuroinflammation, the NAD^+^ level is elevated in APCs, causing a measurable net increase in NAD^+^ content in the CNS; however, at the same time, the adjacent tissue is starved from NAD^+^, causing the neurons to be particularly vulnerable to various oxidative and neuroexcitatory insults, resulting in neurodegeneration due to NAD^+^ deficiency and subsequent bioenergetic collapse and ultimately cell death. The excessive activation of IDO on one hand can deplete the extracellular TRP—a precursor to NAD^+^—on the other hand, it can increase intracellular NAD^+^ levels in immune cells. IFN can enhance the viability and endurance of astrocytes against oxidative stress via the increasing intracellular NAD^+^ levels [247,269,270,271]. The increment in intracellular NAD^+^ mediated by IFN-γ is abrogated by the inhibition of IDO-1. Additionally, stimulation with IFN-γ along with the concomitant inhibition of PARP-1 results in increased NAD^+^ production in glia cells and macrophages [247,269,270,271]. IFN-γ possesses potent antiviral and antimicrobial activities, which were found to completely rely on its ability to induce IDO-1 activity in various immune cells [272,273,274,275,276,277].

#### 4.5.6. Treating MS in Light of Kynurenines

As described in detail above, kynurenines are important modulators of the immune system, redox reactions, and excitotoxicity. Different members of the pathway exert opposite effects; thus, some induce neurodegeneration, while others promote neuroprotection. It is therefore rational to think that by modulating this pathway, these effects can be harnessed to develop new therapies in a number of diseases, including MS.

In the past decades, several promising pre-clinical data emerged regarding kynurenines and its analogs as therapeutic targets in experimental models of MS, showing potential benefits. Their in-depth, detailed description, however, exceeds the scope of this study [8]. From many potential candidates, we would like to highlight one molecule, laquinimod, which is of particular interest. In addition to showing remarkable structural similarity to KYNA, it was investigated in several human clinical studies for a number of conditions, including MS. In the next paragraphs, we aim to summarize the mechanism of action and effect on the immune system and potential neuroprotective attributes of laquinimod.

##### Laquinimod

**Figure 2:**
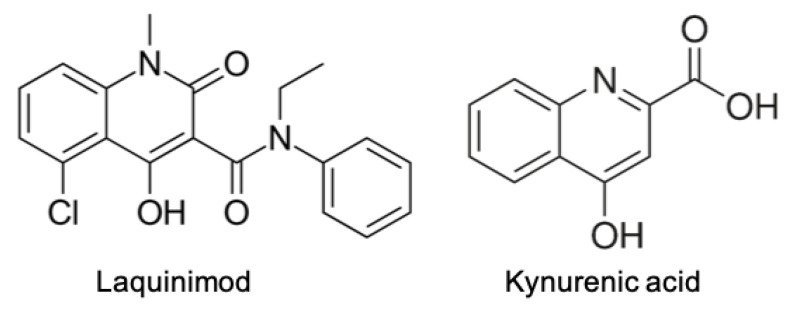
The chemical structure of laquinimod and kynurenic acid.

Laquinimod (or N-ethyl-N-phenyl-5-chloro-1,2-dihydro-4-hydroxy-1-methyl-2-oxo-3-quinolinecarboxamide) (Figure 2) was first synthesized as a structural variant of roquinimex, an initially promising, but later retracted drug for MS (due to severe adverse effects) [278,279]. It displays a remarkable structural similarity to kynurenines, with proposed immunomodulatory and neuroprotective attributes, rather than immunosuppressive effects. It is almost completely (98%) bound to proteins in the plasma, with the ability to diffuse freely across the BBB [280,281]. Estimations put its concentration in the CNS around 13% of the blood concentration [282]. It has a half-life of approximately 80 h and it is metabolized by cytochrome P450 (CYP 450) enzyme family [279].

Even though laquinimod’s mechanism of action is still not clear, several studies have shown its strong capabilities to guide the immune system toward the anti-inflammatory paths. In vitro studies established that laquinimod reduces the expression of MHC class II genes and alters the expression of genes involved in the activation of T and B cells [283]. Seemingly it also increases the CD86^+^ CD25^+^ and IL10^+^ CD25^+^ immunoregulatory subpopulations of B cells, in turn reducing the proliferation and the percentage of INFγ^+^ T cells [284].

Animal models demonstrated that laquinimod blocks the release of inflammatory cytokines (TNFα and IL-1) in monocytes and hinders the adhesion and migration of leukocytes by decreasing levels of matrix metalloproteinase 9 (MMP9) and very late antigen-4 (VLA-4) [285,286,287]. Additionally (by a dose-dependent effect), it inhibits the migration of T cells into the CNS and promotes the production of anti-inflammatory cytokines (TGFβ and IL-4) instead of pro-inflammatory ones (TNFα and IL-12) [281]. It reduces the number of CD4^+^ dendritic cells and through it, the amount of Th1 and Th17 cells, while increasing the number of Treg cells [288]. Laquinimod was also shown to decrease microglial activation, leading to reduced axonal damage [279]. A recent study has concluded that laquinimod can induce remyelination through the protection of oligodendrocyte progenitors during differentiation [289].

Human data are convincing as well, as decreased secretion of chemokines by mature dendritic cells and reduced number of CD1c^+^ and plasmacytoid CD303^+^ DCs in the peripheral blood were found in patients treated with laquinimod [290]. Laquinimod also inhibits T cell secretion of INF-γ, IL-17, granulocyte-macrophage colony-stimulating factor, and TNF-α, while increasing the production of IL-4 by CD4^+^ T cells [288,290].

However, laquinimod is seemingly not only capable of modulating the immune system, but has neuroprotective properties as well. By increasing the level of brain-derived neurotrophic factor—a protein essential to the development of the CNS—it regulates synaptic plasticity and enhances neuronal and axonal growth [291]. BDNF overexpression was found in the striatum, lateral septal nucleus, nucleus accumbens, and the cortex of EAE mice after laquinimod treatment. Additionally, an increased amount of immunosuppressive Foxp3^+^ Treg cell was observed, which was associated with reduced astrogliosis, axonal, and myelin damage [292]. In a study evaluating blood samples of 203 MS patients treated with laquinimod a significant increase in serum levels of BDNF was seen in 76% of the patients [293]. Evidence from animal studies suggests that laquinimod may suppress inducible nitric oxide synthase activity; therefore, it can decrease the level of nitric oxide, a potent neurotoxin [287]. 

Two pivotal, phase III clinical studies were conducted with laquinimod in MS. The ALLEGRO study was placebo-controlled; the BRAVO study had an active comparator (IFN-β i.m.) and placebo arm. In summary: the drug showed a modest effect on the annualized relapse rate (21% in the BRAVO study, 0.30 ± 0.02 vs. 0.39 ± 0.03, *p* = 0.002 against placebo in the ALLEGRO study) [294,295]. A significant reduction in disability progression (40.6%; *p* = 0.042 in the BRAVO, 11.1% vs. 15.7%, *p* = 0.01 against placebo in the ALLEGRO study) was observed [294,295]. Brain atrophy rates were significantly reduced against placebo in the BRAVO study (treatment effect 0.28%, *p* < 0.001) [295]. There was some controversy regarding MRI endpoints: in the ALLEGRO study, laquinimod showed a significant reduction in Gd^+^ T1-lesion number and the number of new or enlarging T2-lesions (*p* < 0.001), while in BRAVO, it showed no significant benefits [294,295]. The safety profiles were consequently favorable throughout the studies.

Despite this, the Committee for Medicinal Products for Human Use (CHMP) did not approve laquinimod in 2014. The CHMP reasoned that during the animal studies, chronic laquinimod exposure raised the occurrence of malignancies, and even though no malignancies were recorded during human trials, it was deemed too high a risk. Furthermore, it also showed teratogenic tendencies. Combined with the relatively modest effectiveness of the drug, they felt the potential risk of chronic exposure outweighs the benefits of disability reduction; thus, they rejected approval.

In summary, we can conclude that laquinimod has a dual effect by modulating the immune system and—probably even more importantly—shows strong neuroprotective attributes. In spite of these strengths, as described above, it was not approved by the CHMP. However, there are some data that laquinimod may be a beneficial add-on therapy in the future for MS. Additionally, as studies are still ongoing in other diseases—e.g., Huntington’s disease—and there are strong pre-clinical results in hemorrhagic stroke, laquinimod may still become an important neuroprotective therapy in the near future [279,296].

## 5. Conclusions

Alterations can be seen in several cascades and pathways involved in immunomodulation, redox mechanisms, energy management, and the upkeep of genomic integrity of a cell in several neurodegenerative diseases, including MS. This results in diminished resistance and increased vulnerability of neuronal cells to oxidative stress and excitotoxicity. In the past years, intensive research has been invested into the kynurenine system; thus, our understanding of the roles of the metabolites and enzymes participating in the KP has been expanded considerably. Even so, many unsettled issues and controversies remain related to their precise function in immune modulation, neuroprotection, and excitotoxicity. Nonetheless, with recent advances confirming the kynurenine system’s pivotal role in the pathogenesis of neurodegenerative diseases, it is highly likely that in the near future additional physiological and pathological roles for neuroactive kynurenines might be unearthed. Additionally, they might prove to be promising targets for drug development based on already known and their yet to be discovered roles. With the rise of targeted drug development and genetic engineering, overcoming past obstacles might indeed become a reality. Targeting the KP in various sites with new, specific agents may be achieved, making the prevention and treatment of several diseases possible by appropriate pharmacological or genetic manipulations.

## Figures and Tables

**Table 1:** Major binding sites and actions of kynurenic acid.

Receptor	Ligand	Action	IC/EC50	Effect
GPR35	cGMP, LPA, T3, rT3, DHICA	Agonist	1–10 µM [48,49,50,51,52]	hyperpolarisation, adenylate cyclase inhibition
AHR	Xenobiotic chemicals	Agonist	10-100 µM [53,54,55]	migration, proliferation, immunmodulation
NMDAR	Glycine, D-serine (glycine-2 co agonist NR1 site)	Antagonist	~8–10 µM [56,57,58,59,60]	excitation, plasticity, neurodegeneration, depolarization, Ca^2+^ influx
NMDAR	Glutamate, NMDA (glutamate/NMDA NR2 site)	Antagonist	~200–500 µM [57,58,60,61,62,63,64,65]	excitation, neurodegeneration, depolarization, Ca^2+^ influx
AMPA/ Kainate	Glutamate	Antagonist	~250 µM [57,58,64,65,66]	excitation, depolarization
Free radicals	n/a	n/a	>200 µM [67,68]	hydroxyl, superoxide radical complexation

Abbreviations: GPR35, G protein-coupled receptor 35; AHR, aryl hydrocarbon receptor; NMDAR, N-methyl-D-aspartic acid receptor; AMPA, α-amino-3-hydroxy-5-methyl-4-isoxazolepropionic acid; cGMP, cyclic guanosine monophosphate, LPA, lysophosphatidic acid, T3, triiodothyronine, rT3, reverse triiodothyronine, DHICA, 5,6-dihydroxyindole-2-carboxylic acid. IC/EC50, half maximal inhibitory concentration and half maximal effective concentration respectively.

## Abbreviations

3-HAO	3-hydroxyanthranilate oxidase
3-HK	3-hydroxykynurenine
AHR	aryl hydrocarbon receptor
AMPA	α-amino-3-hydroxy-5-methyl-4-isoxazolepropionic acid
ANA	anthralinic acid
APC	antigen presenting cell
ATP	adenosine triphosphate
BBB	blood-brain barrier
CDxx	cluster of differentiation xx
CHMP	Committee for Medicinal Products for Human Use
CNS	central nervous system
DC	dendritic cell
EAE	experimental autoimmune encephalitis
GRP35	G-protein coupled receptor 35
IDO 1,2	izoenzymes indoleamine 2,3-dioxygenase 1 and 2
IFN-β	interferon beta
IFN-γ	interferon gamma
IL	interleukin
KAT 1, 2	kynurenine aminotransferase 1 and 2
KMO	kynure nine 3-monooxygenase
KP	kynurenine pathway
KYN	kynurenine
KYNA	kynurenic acid
MMP9	matrix metalloproteinase 9 (MMP9)
MP	methoxyndole pathway
MS	multiple sclerosis
NAD^+^	nicotinamide adenine dinucleotide
NADP^+^	nicotinamide adenine dinucleotide phosphate
NaMN	nicotinic acid mononucleotide
NAS	N-acetylserotonin
NK	natural killer cell
NMAPT	nicotinamide phosphoribosyltransferase
NMDA	N-methyl-D-aspartate
NMDA	N-methyl-D-aspartate receptor
NMN	nicotinamide mononucleotide
NMNAT	nicotinamide mononucleotide adenyl transferase
PARP	poly(ADP-ribose) polymerase
PBMC	peripherial blood monocytes
PMN	polymorphonuclear cells
PPMS	primary progressive multiple sclerosis
QUIN	quinolinic acid
RRMS	relapsing-remitting multiple sclerosis
SS	Sjögren’s syndrome
TDO	tryptophan 2,3-dioxygenase
TGF-β	transforming growth factor beta
TNFα	tumor necrosis factor alpha
Treg	regulatory T cell
TRP	tryptophan
VLA-4	very late antigen-4
